# Efficient Entrapment of Alpha-Synuclein Biotinylated Antibody in KCC-1-NH-CS_2_ and Application for the Sensitive Diagnosis of Parkinson’s Using Recognition of Biomarker: An Innovative Electrochemical Label-Free Immunosensor for the Biomedical Analysis of Neurodegenerative Diseases

**DOI:** 10.3390/bios12100911

**Published:** 2022-10-21

**Authors:** Hossein Navay Baghban, Mohammad Hasanzadeh, Yuqian Liu, Farzad Seidi

**Affiliations:** 1 Jiangsu Co-Innovation Center for Efficient Processing and Utilization of Forest Resources and International Innovation Center for Forest Chemicals and Materials, Nanjing Forestry University, Nanjing 210037, China; 2 Pharmaceutical Analysis Research Center, Tabriz University of Medical Sciences, Tabriz 5165665811, Iran; 3 Nutrition Research Center, Tabriz University of Medical Sciences, Tabriz 5165665811, Iran

**Keywords:** immunosensor, alpha-synuclein, encapsulation, biomedical analysis, ceramic material, immunocomplex, biotechnology, neurodegenerative disease, Parkinson

## Abstract

The early detection of Parkinson’s disease (PD) is a critical issue in terms of efficiency. Alpha-synuclein (α-Syn) is a biomarker in PD checks. Alpha-synuclein (α-syn) is the major constituent of Lewy bodies and a pathogenic hallmark of all synucleinopathies, including PDs, dementia with Lewy bodies, and multiple system atrophy. In this study, KCC-1-NH-CS_2_ was conjugated with biotinylated Ab and entrapped in P(β-CD) polymer cavities. Using this approach, a novel electrochemical label-free immunosensor was designed for the quantification of α-syn in real human samples. For this purpose, the glassy carbon electrode electropolymerized with P(β-CD) biopolymer provided an excellent matrix for entrapping of KCC-1-NH-CS_2_ loaded with the biotinylated antibody of α-syn. Using the chronoamperometric technique, the proposed immunosensor shows a suitable range of 0.02 to 64 ng/mL for the determination of α-syn. Additionally, a low limit of quantification of the engineered biosensor was obtained at 0.02 ng/mL. The developed immunosensor’s adequate stability, sensitivity, and selectivity, together with its ease of manufacture, make it a promising diagnostic technique for further research. This study also will pave the way for further applications of the synergetic effect of β-CD and KCC-1-NH-CS_2_ for biomedical analysis in the near future.

## 1. Introduction

Parkinson’s disease (PD) is a common neurodegenerative illness among the elderly that can be caused by a variety of factors [1]. There is no specific and reliable diagnostic PD check [2]. What makes this more challenging is the difficulties of differential diagnosis for Parkinson’s disease, given that some symptoms may overlap with those of other diseases, particularly at the early stages of PDs [3,4]. So, tracing biomarker abnormalities can be of good value for diagnosis and therapy. Numerous research has shed light on the significance of α-syn and other biomolecules in this regard. From an analytical point of view, the amount of α-syn in normal human plasma was determined to be around 16.5 ng/mL.

As a result, testing for ultra-trace levels of α-syn has a high potential for the early detection of PDs, as accurately defining biomarkers is critical for Parkinson’s disease control and treatment. Numerous methods for quantifying α-syn have been utilized, including chromatography, ELISA, Western blotting, and serology. Even though these approaches yield accurate test results, they must overcome various obstacles, such as expensive and sophisticated equipment, experienced technicians for performing and interpreting tests, and a low level of specificity and high sensitivity. Because of this, the demand for a simple, effective, and reliable method, such as sensors and biosensors, has grown [5,6,7].

Sensors and biosensors have recently been devised as analytical tools for the recognition of a broad array of molecules. They have been devised in different types, the most important of which are immunosensors. Identifying antibodies/antigen complexes is the basis of immunosensors’ function [8,9,10]. This recognition has long been applied in the enzyme-linked immunosorbent assay (ELISA) and has been a staple of medical laboratories for over thirty years. For a variety of reasons, antibodies are an essential class of detection markers.

One important type of immunosensors is label-free immunosensors, as label-free immunosensors with simple antigen recognition avoid time-consuming preparation techniques and enable a simple assay with quick detection and inexpensive assay cost. So, we have aimed to create a sensitive direct electrochemical α-syn bioassay using label-free immunosensor technology.

Many electrochemical bioanalysis experiments use glassy carbon electrodes (GCE) as an excellent substrate with good chemical stability and great electrical conductivity. It is highly advised that the electrode’s surface be modified with natural polymers such as β-CD to optimize its function [11,12,13,14,15]. Cyclodextrins (CDs) are cyclic oligosaccharides consisting of 1,4-linked-D-glucopyranoside units. There are three types of varying molecular cavity sizes: *α*-CD (6 units), β-CD (7 units), and *γ*-CD (8 units). This type of confirmation results in a preference for certain host structures. The molecular truncated cone structure of CDs is distinguished by the presence of two hydroxyl groups: the smaller rim contains primary hydroxyl groups, while the larger rim contains secondary hydroxyl groups. There are six-membered glucopyranoside units in the cone that include CH groups and glycosidic oxygen, which provide its hydrophobic character in addition to the hydrophilic top and bottom cavities. It is possible to construct novel supramolecular complexes with a variety of fascinating compounds since they are hydrophobic inside and hydrophilic outside, coupled with the existence of two kinds of hydroxyl groups in their construction. Because of this, CDs have received a lot of attention from chemists in a variety of areas, such as biosensor technology [16,17].

Additionally, porous materials offer a broad range of applications in material science, serving as remarkable building blocks for the advancement of several technologies [18]. Silica’s usage as a porous material is widely established due to its advantageous qualities such as low density, low toxicity with high biocompatibility, ease of surface modification, stability, and low cost [19].

Morphology-controlled nanoparticles such as silica are promising materials in the development of technology to solve energy, environmental, and health concerns. Dendritic fibrous nano-silica (DFNS), like KCC-1, is one of the most noteworthy inventions [19]. Unlike other silica materials, this one has a fibrous shape rather than a tubular porous structure. It possesses a large surface area, adjustable pore size and pore volume, customizable particle size, and excellent stability [19].

We developed a novel α-syn electrochemical immunosensor due to the advantageous features of poly (β-CD) and KCC-1. In this study, KCC-1-NH-CS_2_ nanoparticles were synthesized and tested for their ability to encapsulate monoclonal antibodies on the surface of modified P (β-CD) modified GCE. Poly (β-CD) was used as a biocompatible and conductive matrix that entrapped high loads of KCC-1-NH-CS_2_ conjugated with biotinylated Abs. Notably, an eco-friendly electrodeposition method was used for the electropolymerization of β-CD on GCE. For the first time, KCC-1-NH-CS_2_ was successfully employed to provide a large surface area of contact to the antibody, and as a result, the peak current of the immunosensor was boosted significantly, which was mainly because of its shape and functional groups. Moreover, the reproducibility and stability of immunoassay were more than acceptable by in the tests carried out.

## 2. Experimental

### 2.1. Chemicals and Reagents

Bovine Serum Albumin (BSA), potassium ferrocyanide K_4_Fe (CN)_6_, potassium ferricyanide K_3_Fe (CN)_6_, N-hydroxysuccinimide (NHS, 98.0%), and 1-ethyl-3 carbodiimide-(3-dimethyl aminopropyl) (EDC, 98.0%) were purchased from Sigma-Aldrich. Phosphate-buffered saline (PBS) solution (0.05 M) was prepared by Na_2_HPO_4_ (0.1 M) and NaH_2_PO_4_ (0.1 M) with 0.15 M of NaCl in deionized water. Human Alpha-synuclein ELISA kit (CanAg/PSA EIA) involving varied concentrations of the alpha-synuclein antigen, biotinylated antibody, and standard buffer and wash solutions were purchased from ZellBio GmbH (Berlin, Germany). Deionized water was purchased from Shahid Ghazi pharmaceutical Company (Tabriz, Iran). To make the stock solution of β-CD (6 mM), 0.0567 g was mixed with required amount of PBS buffer (0.05 M and pH = 4) and stored at 4 °C.

### 2.2. Apparatus

The GCE surface was electrochemically modified in a common three-electrode cell (from Metrohm, Barendrecht, Netherland) operated by an electrochemical system that included an AUTOLAB system with PGSTAT302 N. (Eco Chemie, Utrecht, The Netherlands). The system was run using the NOVA 1.11 software. Electrochemical assays were carried out on an electrochemical system that included a PalmSens system with PS4.F1.05 and a typical three-electrode cell (Metrohm) with a GCE that was from Azar Electrode Co. (Urumia, Iran) as the working electrode (d = 2 mm), Pt wire as the counter electrode, and Ag/AgCl saturated KCl as the reference electrode (Palm Tools, Utrecht, The Netherlands). The PSTrance 5.3 program was used to run the system on a PC. The amplitude of the ac voltage utilized was 10 mV. The CV and ChA techniques were carried out to modify the surface and α-syn monitoring in standard samples, respectively. The DPV technique was also used for both selectivity and repeatability tests. The surface morphology of the modified electrodes was studied using a high-resolution field emission scanning electron microscope (FE-SEM, Hitachi-SU8020, Praha, Czech) scanning at 3 kV.

### 2.3. KCC-1-NH-CS_2_ Synthesis

KCC-1 and KCC-1-NH_2_ were synthesized following the technique described in our prior research [18]. Dithiocarbamate functionalized KCC-1-NH_2_ as follows: 100 mg of CS_2_ was dissolved in 20 mL of acetonitrile containing 1 percent Et_3_N and 90 mg of KCC-1-NH_2_. The resulting mixture was swirled overnight to ensure complete dissolution. Finally, an evaporator was used to dry the solution. Characterization of this advance silica mesoporous materials were performed by TEM (Figure S1, Supporting Information)), FESEM (Figure S2), and EDS/FT-IR (Figure S3) methods.

### 2.4. Encapsulation of the Antibody into KCC-1-NH-CS_2_

KCC-1-NH-CS_2_ with NH and CS_2_ functional groups provides two active sites for immobilizing Ab on the surface of GCE; to accomplish this, after dissolving 0.001 g of KCC-1 in 100 μL of phosphate buffer, 300 μL of biotinylated antibody was added to the solution and shaken for 24 h at room temperature. After this period, the solution was centrifuged for 5 min at 6000 rpm, and the supernatant was separated from the sediment with a sampler before being kept at 4 °C.

### 2.5. Preparation and Decontamination of the Electrodes

In order to make modifications, the bare GCE had to be thoroughly cleaned both mechanically and electrochemically. For mechanical cleaning, electrodes were polished on a soaked velvet cloth for 10 min, followed by 10 min of sonication in 1:1 water/acetone. For the electrochemical procedure, electrodes were immersed in H_2_SO_4_ (0.1 M) and CV technique for 10 cycles, and a scan rate of 0.1 V/s between 0 and 1 V was applied.

### 2.6. Electropolymerization of Beta-Cyclodextrin on the Surface of GCE

Electropolymerization is a straightforward, environmentally friendly, and effective method of synthesizing polymers. The polymers formed using this approach do not contain any catalysts or oxidant fragments, and their physical and chemical characteristics are determined by the electrochemical conditions used during formation [20]. Several articles have been published on the development of biosensors based on the electrodeposition of β-CD on the surface of a GCE, which shows the popularity of this method for the construction of biosensors [21,22,23,24,25]. 

To commence electropolymerization, the monomer must first be oxidized to generate a radical cation. The two oxidized monomers would then combine to create a dimer. The resulting dimer would be oxidized to generate a radical cation. This oxidized dimer can create a trimer when combined with another oxidized monomer. Finally, a growing polymer chain is made when monomer units are placed at the active site in sequential order. The radical cations could be linked by carbon–carbon ties, nitrogen–nitrogen, as well as carbon–nitrogen [26].

To achieve a homogeneous coating of P(β-CD) on the surface of the GCE electrode, the electropolymerization of β-CD was carried out on the clean electrode by immersing it in a 6 mM β-CD in PBS solution and applying CV for 40 cycles between −2 and +2 V (Ag/AgCl) (Figure S4). After this operation, two anode peaks and one cathode peak appeared, as illustrated in the diagram. More specifically, the current grew rapidly until about the 12th cycle, then lessened in intensity in subsequent cycles, signaling that the film had possibly achieved its maximum depth [27]. Not only was the cathode peak distinguishable, but it also had great strength, implying that the electropolymerization was likely successful, as experimental investigations demonstrate that the cathode peak is closely associated with successful synthesis [26]. After rinsing the β-CD-coated GCE with deionized water, it was prepared for the following steps.

### 2.7. Immunosensor Assembly

**Scheme 1:**
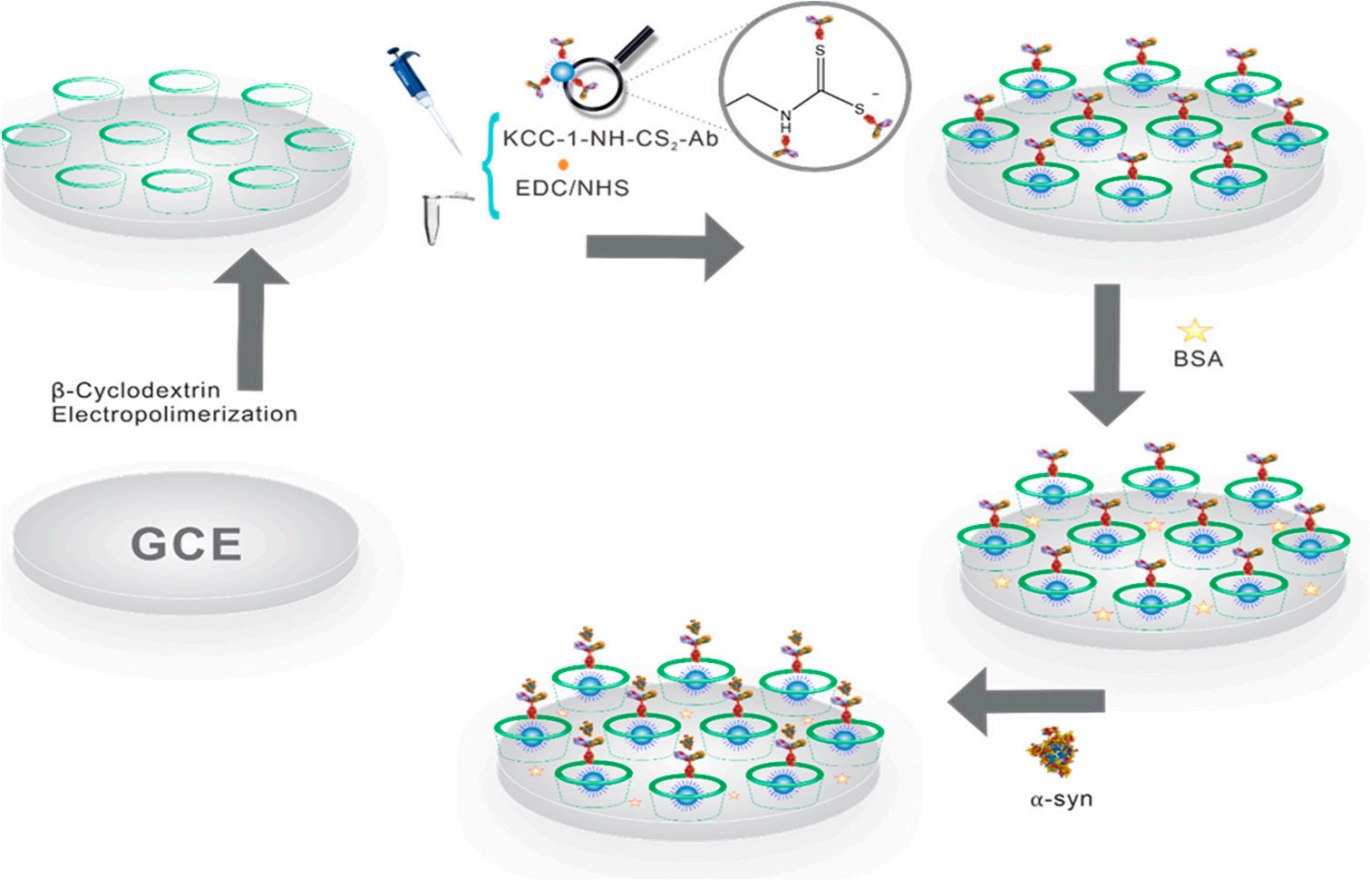
The fabrication procedure of the electrochemical immunosensor for the recognition of α-syn biomarker.

For the preparation of the immunosensor, first, Abs entrapped in KCC-1-NH-CS_2_ was activated by a solution of EDC/NHS (0.2 M) in a 1:1 *v*/*v* ratio for 20 min. Then, 5 µL KCC-1-NH-CS_2_-Ab was dropped carefully on the GCE-P(β-CD) and kept at 4 °C for 6 h. The incubated electrode was then rinsed with deionized water to remove any unbound anti-alpha-synuclein. The next step was incubation with BSA for 5 min, as it can block the unspecific bindings. Finally, the resulting label-free immunosensor was rinsed with deionized water to be subjected to an immunochemical reaction with various α-syn concentrations in standard samples. Scheme 1 illustrates the fabrication steps of the aforementioned immunosensor.

## 3. Results and Discussion

### 3.1. The SEM Characterization of the Modified GCE

FE-SEM images of GCE-P(β-CD), GCE-P(β-CD)–KCC-1-NH-CS_2_-Ab, and GCE-P(β-CD)–KCC-1-NH-CS_2_-Ab-BSA-Ag are represented in Figure S5. Figure S5A indicates that the electropolymerization of β-CD on the surface of GCE has been completed successfully, and macromolecules of β-CD, with an arranged and orderly interwoven structure, were formed. This even layer, which consists of compact particles ranging from 35 to 40 nm in size, provides a huge platform for the trapping of KCC-1-NH-CS_2_-Ab molecules. After incubation of KCC-1-NH-CS_2_-Ab, the morphology of the GCE-P(β-CD) surface has changed noticeably, and the aggregation of KCC-1-NH-CS_2_-Ab in cubic shapes confirmed the successful process of KCC-1-NH-CS_2_-Ab entrapment by β-CDs’ cavity (Figure S5B). Finally, the transformation of cubic to a spherical form (Figure S5C) by incubation of α-syn antigen after adding BS is a clear sign of a successful Ab-Ag immunocomplex.

### 3.2. Electrochemical Properties of the Immunosensor’s Surface

**Figure 1:**
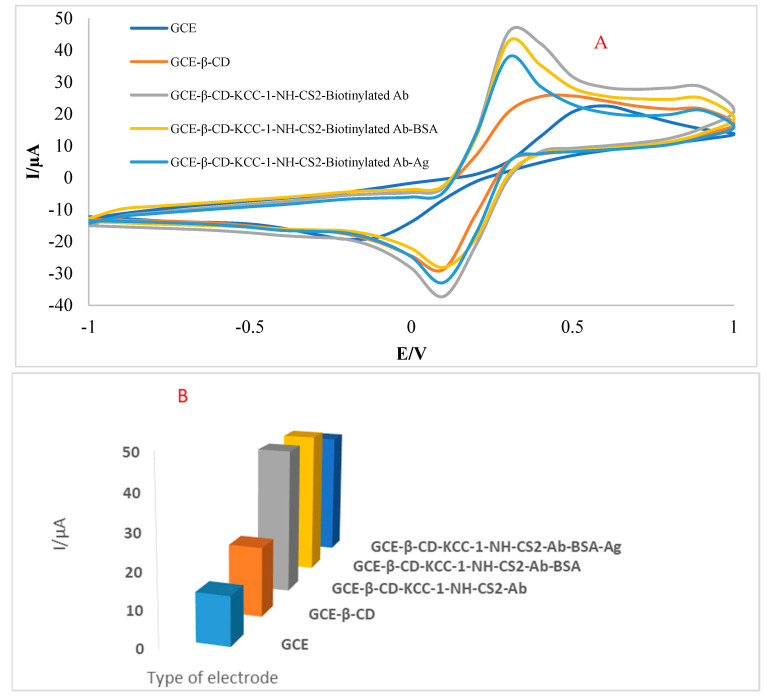
(**A**) CV characterization of step-by-step fabrication of immunosensor, the supporting electrolyte was 0.05 M solution [Fe (CN)_6_]^3−/4−^/KCl with a potential range from −1 to 1, scan rate = 0.1 V/s. (**B**) Comparison of peak currents recorded in the process of immunosensor fabrication.

To investigate the electrochemical characteristics of the modified electrode surface, the immunosensor’s development was analyzed step-by-step by cyclic voltammetry (CV) technique using [Fe (CN)_6_]^3−/4−^ as a supporting electrolyte in the potential range of −1 to 1 V versus Ag/AgCl. After electropolymerization of GCE with β-CD, the increase of 5882 µA in the anodic peak current was noticeable, which indicates the redox reaction of Fe (III)/Fe (IV) at the polymeric interface due to the compact layer of β-CD formed at the GCE substrate. The main reason for this increase in a redox reaction is the existence of functional groups in the P(β-CD) layer. Another change is the shift of peak position towards negative potential, which can be attributed to the successful coating of P(β-CD). By adding KCC-1-NH-CS_2_-Ab, the peak current doubled and reached just over 40 µA. This could be mainly because of the high load of KCC-1-NH-CS_2_-Ab on the P(β-CD) layer as well as the high load of entrapped Ab on KCC-1-NH-CS_2_. We also had a shift of peak position for the second time, verifying successful Ab immobilization. As BSA was used for minimizing the non-specific binding after incubation with it for 5 min, the peak current decreased minimally. The prepared immunosensor was subjected to α-syn, and a moderate decrease in peak current confirmed successful binding with entrapped Abs on the surface of the electrode (see Figure 1 and Table 1 for more information). 

### 3.3. Analytical Characteristics of the Immunosensor

**Figure 2:**
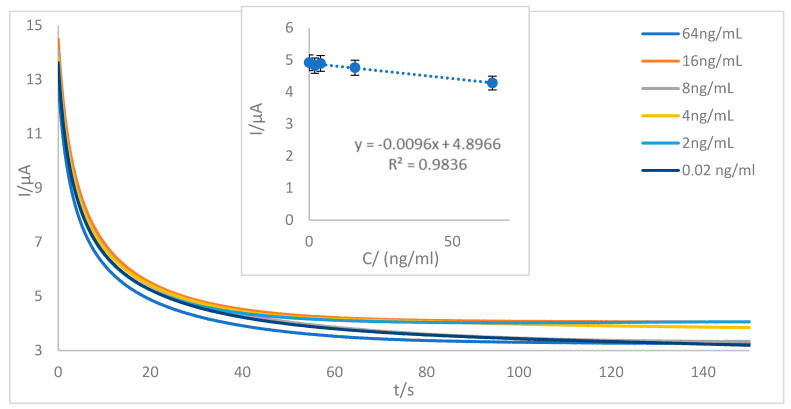
ChA curves of the GCE-P(β-CD)-KCC-1-NH-CS_2_ in different concentrations of α-Syn (0.02, 2, 4, 8, 16, 64 ng/mL). The supporting electrolyte is 0.01 M ferricyanide/ferrocyanide. The applied potential was 0.3 V vs. Ag/AgCl, and the record time was set to 140 s. Inset: related calibration curve (n = 4, SD = 2.44).

For the determination of the candidate biomarker, different concentrations of the target analyte (α-syn, in the range of 0.02 to 64 ng/mL) were tested (Figure 2) by dropping on the surface of the designed immunosensors. Then, they were immersed in an electrochemical cell containing 0.01 M ferricyanide/ferrocyanide for electrochemical measurements. Among all electrochemical techniques, ChA yielded the best result for the analytical approach, and at low concentrations of α-syn, the developed immunosensor proved to be extremely sensitive. According to this study, by increasing the concentration of α-syn, the peak current experienced a drop, which was in a linear relationship with 0.02–64 ng/mL of the analyte. According to the calibration curve, in this range of concentration, as shown in Figure 2, the linear regression equation was acquired as follows:

I_p_/µA = −0.0096C _(α-syn)_ (ng/mL) + 4.8966, (R^2^ = 0.9836)

By looking back to reports for the α-syn biosensors (Table 2), it is noticeable that the most common receptor was an antibody. This is because of the simplicity of production and use of this natural receptor. Additionally, the Ag-Ab immunoreaction gives high sensitivity to biosensors. These, coupled with the reliability of antibody usage, were compelling reasons for deploying Ab as a bioreceptor in this work. Other published works have almost used metal nanoparticles for signal amplification; nevertheless, in this work, we used KCC-1-NH-CS_2_ as an innovative and simple non-metal signal amplifier. This, in turn, gave this analytical approach the edge in terms of simple fabrication. Compared with most other biosensors in the table, the proposed immunosensor enjoys a wider linear range of 0.02–64 ng/mL. Moreover, LLOQ for this immunosensor was obtained to be 0.02 ng/mL, which is outstanding among the other previously designed immunosensors for α-syn considering the fact that not only is the devised immunosensor label-free, but it also does not use any metal nanoparticle for signal amplification. To conclude, thanks to these features, the proposed immunosensor can be placed among highly sensitive and reliable biosensors for practical use. 

### 3.4. Effect of Sweep Rate

**Figure 3:**
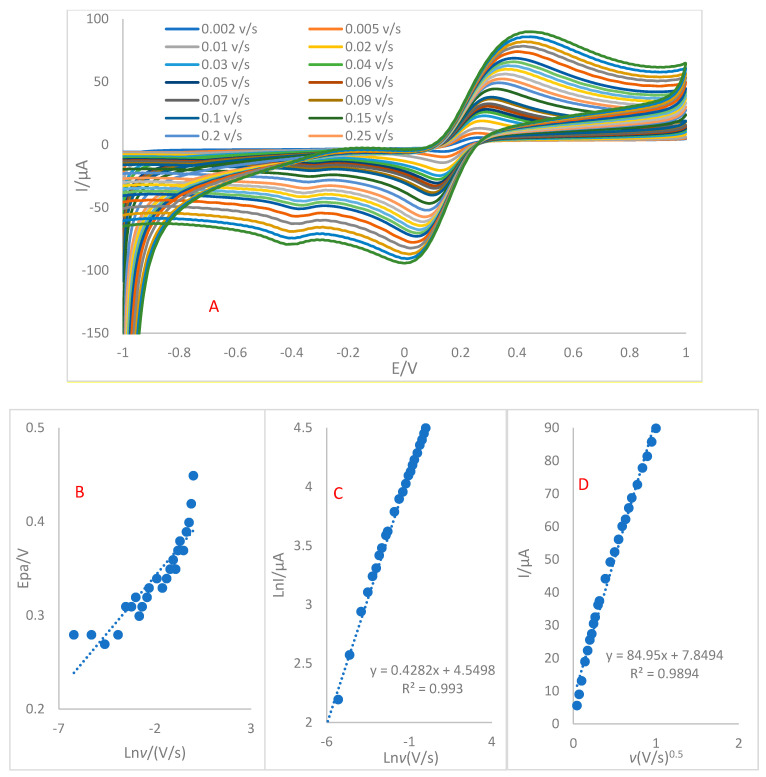
(**A**) CV of GCE-P(β-CD) at scan rates ranging from 0.002 to 1 V/s. (**B**) Dependence of the E_pa_ vs. Ln*v*. (**C**) Dependence of LnI_p_ vs. Ln*v*. (**D**) Dependence of Ip to the square root of sweep rates.

The study of the effect of sweep rate in electrochemical processes can be of great value in understanding electrochemical mechanisms of electron transfer and how mass transport is controlled by either diffusion or adsorption in the electrochemical cells. It also can be helpful for computations of the coefficient of electron transfer (α), coverage of specific areas (Г*), as well as coefficient of electron transfer (α). Therefore, the polymerized sensor’s kinetics were assessed by conducting CV at various sweep rates, ranging from 0.002 to 1 V/s, in the presence of [Fe (CN)_6_]^3−/4−^ (Figure 3). According to the obtained CV graph, a rise in scan rate caused the voltammogram to broaden progressively and the anodic peak current to surge significantly. So, it is highly likely that electrons transfer at the electrode interface, causing the formation of a peak current, which occurs more leisurely at slower scan speeds.

For determining whether the redox process is controlled by adsorption or diffusion, two major factors can be employed: the first one is obtained from the I_p_ *versus v*^1/2^ graph. Using this approach, the intercept of I_p_ *versus v*^1/2^ will show how mass transport is controlled by diffusion at lower sweep rates [36]. The second one is achieved from the Ln Ip versus Ln v graph. In this approach, diffusion control is indicated by a slope of near 0.5, while adsorption control is indicated by a slope of close to 1 of the relationship between lnI_p_ and ln *v* (V/s) [36]. According to the obtained equation: LnI_p_ = 0.4282 Ln *v* + 4.5498 (R^2^ = 0.993), a slope of 0.4282 consolidates the possibility of the diffusion control of the system.

From Figure 3B, it is evident that E_pa_ has a linear relationship with the Napierian logarithm of scan rate, which can be proven to be due to the irreversibility of electrochemical reactions at the modified electrode’s surface. According to the regression equations of Epa = 0.0245 Ln*v* + 0.3909 (R^2^ = 0.8104), increasing the scan rate resulted in a shift of the polymerized electrode’s peak potential to the positive region.

Table S1 (see supporting information) summarizes the electrochemical parameters related to the modified electrode (GCE-P(β-CD)) and compares it with the bare electrode (GCE).

## 4. Analytical Method Validation

### 4.1. Selectivity

The selectivity of the immunosensor is one of the determining factors in its practicality. Therefore, the proposed immunosensor has been subjected to some interferential antigens such as (CYFRA 21.1), (CA15-3), and prostate-specific antigen (PSA). These antigens, as well as α-syn, were incubated on the surface of four prepared immunosensors, and the DPV technique was used for measuring a related peak current in the presence of 0.01 M ferricyanide/ferrocyanide. As shown in Figure S6, the lowest peak current belongs to α-Syn at 40.5545. Knowing that the immunosensor’s response for selective antigen is a signal-off type, α-Syn peak current had been predicted to be the lowest among other antigens, although CA15-3 with the peak current of 44.0412 µA out of these macromolecules can be considered to have the greatest potential interference for engineered immunosensors. These results show that the constructed electrochemical immuno-device has decent selective properties and can distinguish alpha-Syn from other proteins.

### 4.2. Repeatability of Immunosensor

**Figure 4:**
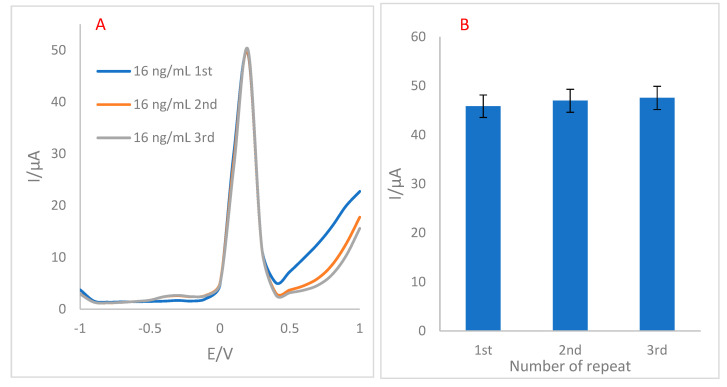
(**A**) DPV of GCE-β-CD–KCC-1-NH-CS_2_-Ab-BSA in the presence of 16 ng/mL α-syn in three repetitions. The supporting electrolyte was 0.05 M solution [Fe (CN)_6_]^3−/4−^/KCl; the potential range was from -1 to 1. (**B**) Comparison of 1st, 2nd, and 3rd peak currents (SD = 2.13, n = 4).

A sensor’s repeatability is an essential indicator for measuring its performance. Repeatability refers to a biosensor’s capacity to provide equal responses under comparable experimental circumstances. For evaluating this factor, the DPV technique was used, and the devised immunosensor underwent an examination in 0.01 M ferricyanide/ferrocyanide three uninterrupted times (Figure 4). From the resulting data, the relative standard deviation (RSD) of 1.49% was calculated. This percentage shows that one of the advantages of the proposed immunosensor can be its great accuracy, which may be because of the proper immobilization of biotinylated Abs on the surface of the electrode.

### 4.3. Inter-Day Repeatability

As the durability of the biosensor over time is important for its practical use, inter-day stability evaluation of the electrode’s substrate was carried out by the CV technique for seven days in a row. For this porous, one electrode was electropolymerized with β-CD and kept at room temperature for 7 days. The electrode underwent CV measurement every day. Recorded voltammograms (Figure S7) revealed relatively high inter-day stability of GCE-P(β-CD). There were steady trivial decreases in the peak current of voltammograms from the first day to the fourth day, followed by an increase on the fifth day. Then, the peak current ended up with a recurrent decrease in the last two days of this period. These data confirmed the stability of P(β-CD) on the surface of GCE for 4 days.

### 4.4. Stability Analysis

As the stability of the β-CD matrix on the surface of the electrode in consecutive measurements can partly indicate the immunosensor’s stability, a GCE-β-CD voltammogram for 100 consecutive cycles has been recorded. From the 1st to the 75th cycle, the peak current experienced a steady increase, which reached the current peak of 37.66 (ΔI = 7.46). Nevertheless, from the 75th cycle to the 100th cycle, there was a slight decrease, and ΔI from the 1st to 100th cycle was obtained to be 6.1636 µA. So, the modified electrode could have the best responses for 75 uses (Figure S8).

## 5. Conclusions

In this study, we devised a simple and reliable label-free immunoassay for sensitive recognition of α-syn. Poly(β-CD) was used as a biocompatible and conductive matrix that entrapped high loads of KCC-1-NH-CS_2_ conjugated with biotinylated Abs. Notably, an eco-friendly electrodeposition method was used for the electropolymerization of β-CD on GCE. For the first time, KCC-1-NH-CS_2_ was successfully employed to provide a large surface area of contact to the antibody, and as a result, the peak current of the immunosensor was boosted significantly, which is mainly because of its shape and functional groups. Moreover, the reproducibility and stability of immunoassay were more than acceptable in the tests conducted. In the analytical approach, the immunosensor performed perfectly in terms of linearity. The linear range and LLOQ for this immunosensor were 0.02–64 and 0.02 ng/mL, respectively. Promisingly, this study can pave the way for the expansion of KCC-1-NH-CS_2_ applications in the fields of electrochemical immunosensor. Moreover, the simplicity used for devising this immunosensor allows for designing miniaturized point-of-care (POC) devices for the early diagnosis of diseases.

## Figures and Tables

**Table 1:** Peak currents and peak positions of GCE and modified GCEs in the process of immunosensor fabrication.

Index	Peak Position	Peak Height
GCE	0.50028	13.4135
GCE-β-CD	0.40028	19.2955
GCE-β-CD-KCC-1-NH-CS_2_-Ab	0.30027	40.9655
GCE-β-CD-KCC-1-NH-CS_2_-Ab-BSA	0.30027	40.8493
GCE-β-CD-KCC-1-NH-CS_2_-Ab-BSA-Ag	0.30027	36.2842

**Table 2:** Comparing our proposed α-syn immunosensor with other α-syn biosensors.

Used Technique	Bioreceptor	Interface of Electrode	LOD/LLOQ (ng/mL)	Linear Range (ng/mL)	Ref.
Photo-electrochemical	Antibody	Au–TiO_2_ NTs	0.034	0.05–100	[28]
EIS, SPR	Aptamer	Thiolated Au	0.001	0.1 nM–0.5 µM	[29]
CV, DPV	Aptamer	Apt-CS-Au	10 pM	60 pM–150 nM	[30]
EIS, CV, SWV	Antibody	Au NP–PGA/ITO	0.135	0.004–2	[31]
EIS, CV	Antibody	PAMAM–Au/C	0.0146	0.02–200	[32]
DPV, EIS	Antibody	CYS/FTO	3.62, 1.13	10–1000	[33]
ChA, EIS	Antibody	AuNCs-graphene	4 (LLOQ)	4–128	[34]
Optical (LSPR)	Chitosan film	AuNPs-chitosan	70 nM	70~700 nM	[35]
ChA	Antibody	P(β-CD)	0.02	0.02–64	This work

## Data Availability

Not applicable.

## Supplementary Materials

The following supporting information can be downloaded at: https://www.mdpi.com/article/10.3390/bios12100911/s1, Figure S1: TEM graphs of KCC-1-NH-CS_2_ in various magnifications; Figure S2. FESEM graphs of KCC-1-NH-CS_2_ (C); Figure S3. (A) EDAX analyses of KCC-1, KCC-1-NH2 and KCC-1-NH-CS_2_. (B) FTIR spectra of KCC-1 and KCC-1-NH-CS_2_; Figure S4. (A) Electropolymerization of 6 mM β-CD in 0.05 M PBS (pH = 4) as a supporting electrolyte on the surface of GCE. The cyclic voltammogram depicts gradual growth of β-CD after 40 successive cycles in the range of −2 to +2 V vs. Ag/AgCl with a scan rate of 0.07 V/s. Inset: three current peaks of β-CD electropolymerization; Figure S5. FE-SEM images of the sequential modified layers on GCE: (A) β-CD, (B) β-CD –KCC-1-NH-CS_2_-Ab (C) β-CD – KCC-1-NH-CS_2_-Ab-BSA-Ag in different magnifications; Figure S6. (A) DPV of GCE-β-CD–KCC-1-NH-CS_2_-Ab-BSA in the presence of α-syn and three other interferences. The supporting electrolyte was 0.05 M solution [Fe (CN)6]3-/4-/KCl; the potential range was from −1 to 1. (B) comparison of α-syn and three other interferences peak current. (SD = 2.28, n = 4); Figure S7. (A) CV of GCE-P(β-CD) in 7 consecutive days. (B) Comparison of 7 consecutive days’ peak current intensity for GCE-P(β-CD) versus time of storage (SD = 1.74, n = 4); Figure S8. (A) CV curves of GCE-P(β-CD) for the 1st, 5th, 10th, 20th, 50th, 75th, and 100th cycles. (B) Dependency of peak currents versus the number of cycles (SD = 1.95, n = 4); Table S1. Electrochemical parameters of GCE-P(β-CD) and GCE (bare electrode); Table S2. Analytical parameters determined for calibration curves of α-Syn using engineered immunosensor.

## References

[1] Siderowf A., Stern M. (2003). Update on Parkinson disease. Ann. Intern. Med..

[2] Dawson T.M., Dawson V.L. (2002). Neuroprotective and neurorestorative strategies for Parkinson’s disease. Nat. Neurosci..

[3] Litvan I., Halliday G., Hallett M., Goetz C.G., Rocca W., Duyckaerts C., Ben-Shlomo Y., Dickson D.W., Lang A.E., Chesselet M.-F. (2007). The etiopathogenesis of Parkinson disease and suggestions for future research. Part I. J. Neuropathol. Exp. Neurol..

[4] Shi M., Bradner J., Hancock A.M., Chung K.A., Quinn J.F., Peskind E.R., Galasko D., Jankovic J., Zabetian C.P., Kim H.M. (2011). Cerebrospinal fluid biomarkers for Parkinson disease diagnosis and progression. Ann. Neurol..

[5] Tao D., Gu Y., Song S., Nguyen E.P., Cheng J., Yuan Q., Pan H., Jaffrezic-Renault N., Guo Z. (2020). Ultrasensitive detection of alpha-synuclein oligomer using a PolyD-glucosamine/gold nanoparticle/carbon-based nanomaterials modified electrochemical immunosensor in human plasma. Microchem. J..

[6] Mobed A., Hasanzadeh M. (2020). Biosensing: The best alternative for conventional methods in detection of Alzheimer’s disease biomarkers. Int. J. Biol. Macromol..

[7] Mobed A., Ahmadalipour A., Fakhari A., Kazem S.S., Saadi G.K. (2020). Bioassay: A novel approach in antipsychotic pharmacology. Clin. Chim. Acta.

[8] Mobed A., Razavi S., Ahmadalipour A., Shakouri S.K., Koohkan G. (2021). Biosensors in Parkinson’s disease. Clin. Chim. Acta.

[9] Mobed A., Hasanzadeh M., Shadjou N., Hassanpour S., Saadati A., Agazadeh M. (2020). Immobilization of ssDNA on the surface of silver nanoparticles-graphene quantum dots modified by gold nanoparticles towards biosensing of microorganism. Microchem. J..

[10] Lequin R.M. (2005). Enzyme immunoassay (EIA)/enzyme-linked immunosorbent assay (ELISA). Clin. Chem..

[11] Hasanzadeh M., Shadjou N. (2016). Electrochemical nanobiosensing in whole blood: Recent advances. TrAC Trends Anal. Chem..

[12] Abdel-Aziz A.M., Hassan H.H., Badr I.H. (2020). Glassy carbon electrode electromodification in the presence of organic monomers: Electropolymerization versus activation. Anal. Chem..

[13] Da Silva L.V., Lopes C.B., da Silva W.C., de Paiva Y.G., dos Santos Silva F.d.A., Lima P.R., Kubota L.T., Goulart M.O.F. (2017). Electropolymerization of ferulic acid on multi-walled carbon nanotubes modified glassy carbon electrode as a versatile platform for NADH, dopamine and epinephrine separate detection. Microchem. J..

[14] Douhal A. (2004). Ultrafast guest dynamics in cyclodextrin nanocavities. Chem. Rev..

[15] Holzinger M., Singh M., Cosnier S. (2012). Biotin—β-Cyclodextrin: A New Host–Guest System for the Immobilization of Biomolecules. Langmuir.

[16] Seidi F., Jin Y., Xiao H. (2020). Polycyclodextrins: Synthesis, functionalization, and applications. Carbohydr. Polym..

[17] Seidi F., Shamsabadi A.A., Amini M., Shabanian M., Crespy D. (2019). Functional materials generated by allying cyclodextrin-based supramolecular chemistry with living polymerization. Polym. Chem..

[18] Abbasy L., Mohammadzadeh A., Hasanzadeh M., Ehsani M., Mokhtarzadeh A. (2020). Biosensing of prostate specific antigen (PSA) in human plasma samples using biomacromolecule encapsulation into KCC-1-npr-NH2: A new platform for prostate cancer detection. Int. J. Biol. Macromol..

[19] Maity A., Polshettiwar V. (2017). Dendritic fibrous nanosilica for catalysis, energy harvesting, carbon dioxide mitigation, drug delivery, and sensing. ChemSusChem.

[20] Imisides M.D., John R., Riley P.J., Wallace G.G. (1991). The use of electropolymerization to produce new sensing surfaces: A review emphasizing electrode position of heteroaromatic compounds. Electroanalysis.

[21] Ansari R., Hasanzadeh M., Ehsani M., Soleymani J., Jouyban A. (2021). Sensitive identification of silibinin as anticancer drug in human plasma samples using poly (β-CD)-AgNPs: A new platform towards efficient clinical pharmacotherapy. Biomed. Pharmacother..

[22] Du D., Wang M., Cai J., Zhang A. (2010). Sensitive acetylcholinesterase biosensor based on assembly of β-cyclodextrins onto multiwall carbon nanotubes for detection of organophosphates pesticide. Sens. Actuators B Chem..

[23] Xie S., Zhang J., Yuan Y., Chai Y., Yuan R. (2015). An electrochemical peptide cleavage-based biosensor for prostate specific antigen detection via host–guest interaction between ferrocene and β-cyclodextrin. Chem. Commun..

[24] Kim G.J., Kim K.O. (2020). Novel glucose-responsive of the transparent nanofiber hydrogel patches as a wearable biosensor via electrospinning. Sci. Rep..

[25] Behyar M.B., Kholafazad-kordasht H., Hassanpour S., Hasanzadeh M. (2022). An innovative electrically conductive biopolymer based on poly (β-cyclodextrin) towards recognition of ascorbic acid in real sample: Utilization of biocompatible advanced materials in biomedical analysis. J. Mol. Recognit..

[26] Pereira A.C., Oliveira A.E.F., Bettio G.B. (2019). β-Cyclodextrin electropolymerization: Mechanism, electrochemical behavior, and optimization. Chem. Pap..

[27] Páramo García U., Ibáñez Cornejo J.G., Batina N. (2011). Electrochemical modulation of the thickness of polypyrrole films by using different anionic dopants. Int. J. Electrochem. Sci..

[28] An Y., Tang L., Jiang X., Chen H., Yang M., Jin L., Zhang S., Wang C., Zhang W. (2010). A photoelectrochemical immunosensor based on Au-doped TiO2 nanotube arrays for the detection of α-synuclein. Chem. Eur. J..

[29] Sun K., Xia N., Zhao L., Liu K., Hou W., Liu L. (2017). Aptasensors for the selective detection of alpha-synuclein oligomer by colorimetry, surface plasmon resonance and electrochemical impedance spectroscopy. Sens. Actuators B Chem..

[30] Taghdisi S.M., Danesh N.M., Nameghi M.A., Ramezani M., Alibolandi M., Hassanzadeh-Khayat M., Emrani A.S., Abnous K. (2019). A novel electrochemical aptasensor based on nontarget-induced high accumulation of methylene blue on the surface of electrode for sensing of α-synuclein oligomer. Biosens. Bioelectron..

[31] Karaboğa M.N.S., Sezgintürk M.K. (2019). Cerebrospinal fluid levels of alpha-synuclein measured using a poly-glutamic acid-modified gold nanoparticle-doped disposable neuro-biosensor system. Analyst.

[32] An Y., Jiang X., Bi W., Chen H., Jin L., Zhang S., Wang C., Zhang W. (2012). Sensitive electrochemical immunosensor for α-synuclein based on dual signal amplification using PAMAM dendrimer-encapsulated Au and enhanced gold nanoparticle labels. Biosens. Bioelectron..

[33] Ge C.-Y., Rahman M.M., Zhang W., Lopa N.S., Jin L., Yoon S., Jang H., Xu G.-R., Kim W. (2020). An electrochemical immunosensor based on a self-assembled monolayer modified electrode for label-free detection of α-synuclein. Sensors.

[34] Aminabad E.D., Mobed A., Hasanzadeh M., Feizi M.A.H., Safaralizadeh R., Seidi F. (2022). Sensitive immunosensing of α-synuclein protein in human plasma samples using gold nanoparticles conjugated with graphene: An innovative immuno-platform towards early stage identification of Parkinson’s disease using point of care (POC) analysis. RSC Adv..

[35] Khatri A., Punjabi N., Ghosh D., Maji S.K., Mukherji S. (2018). Detection and differentiation of α-Synuclein monomer and fibril by chitosan film coated nanogold array on optical sensor platform. Sens. Actuators B Chem..

[36] Faulkner L.R., Bard A.J. (2002). Electrochemical Methods: Fundamentals and Applications.

