# Supplementary material for: Efficient Entrapment of Alpha-Synuclein Biotinylated Antibody in KCC-1-NH-CS2 and Application for the Sensitive Diagnosis of Parkinson’s Using Recognition of Biomarker: An Innovative Electrochemical Label-Free Immunosensor for the Biomedical Analysis of Neurodegenerative Diseases
Source: Biosensors (Basel). 2022 Oct 21;12(10):911. doi: 10.3390/bios12100911 (PMC9599316; doi:10.3390/bios12100911)
Supplement: Supplementary file 1 [file biosensors-12-00911-s001.zip › biosensors-1975699-supplementary.pdf]

Supporting information

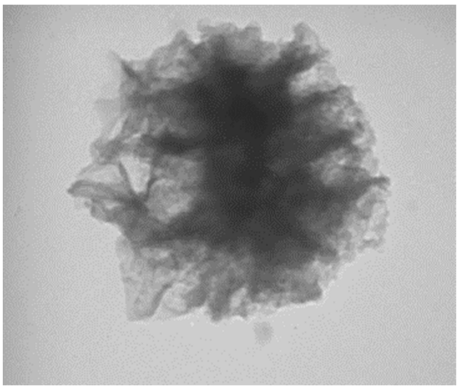

Figure S1. TEM graphs of KCC-1-NH-CS_2 in various magnifications.

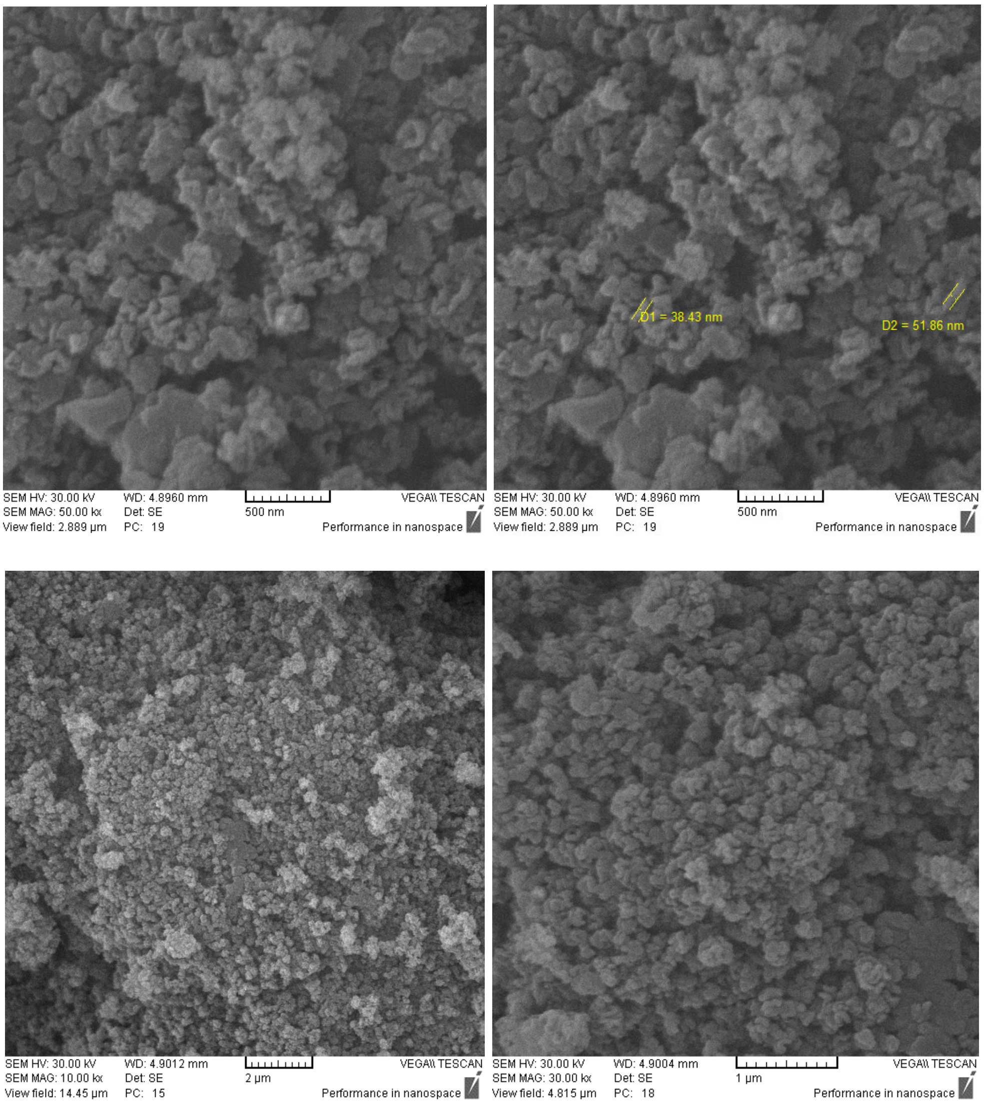

Figure S2. FESEM graphs of KCC-1-NH-CS_2 (C).

Finally, EDAX was carried out for tracing of expected elements in KCC-1, KCC-1-NH_2, and KCC-1-NH-CS_2. As shown in Fig. S3A, KCC-1 mainly consists of O and Si. Functionalizing KCC-1 with NH_2 and after that with CS_2, N elements and S elements increased respectively, which proved the formation of KCC-1-NH-CS_2. The FTIR spectra of KCC-1 and KCC-1-NH-CS_2 are shown in Fig. S3B. The characteristic peaks of the KCC-1 appear at 802 and 1100 cm^{-1} correspond to the symmetric and asymmetric stretching vibrations of the Si-O-Si bond, respectively. New peaks have been observed in the KCC-1-NH-CS_2 spectrum at 1626 cm^{-1} (deformation NH_2), 2520 cm^{-1} (stretching vibration of S-H in mercaptan groups), 2890 cm^{-1} (stretching vibration of aliphatic C-H), and 3400 cm^{-1} (vibration of N-H bond), which confirm the presence of the related functional groups and proves the successful modification of the KCC-1 surface.

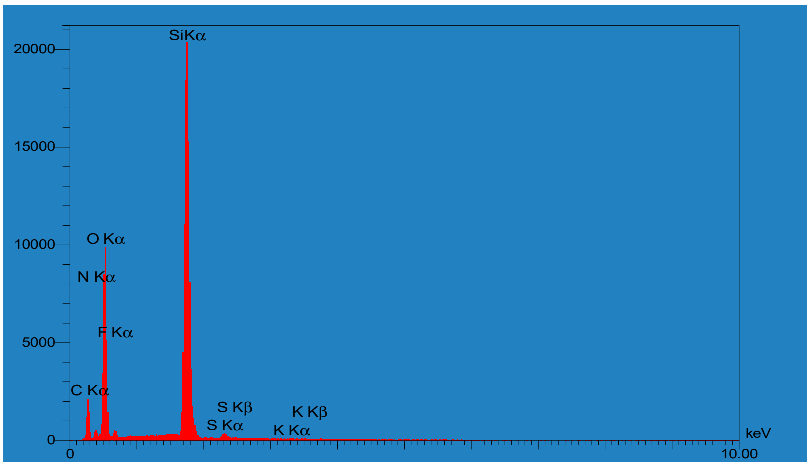

B

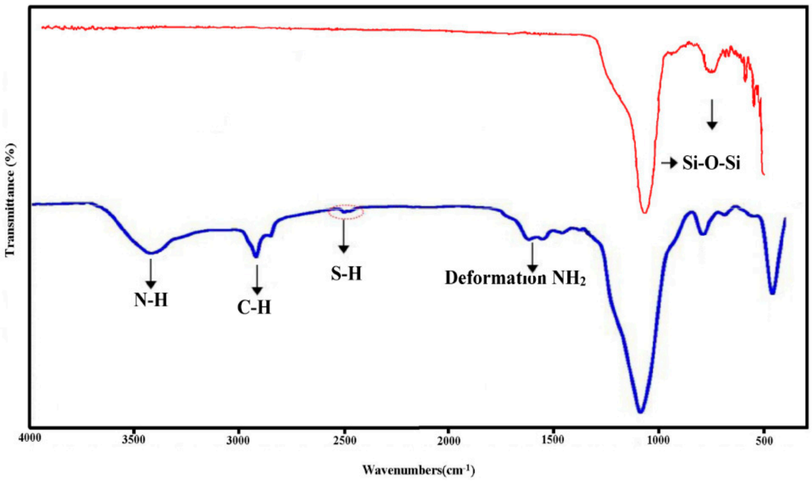

Figure S3. A) EDAX analyses of KCC-1, KCC-1-NH_2 and KCC-1-NH-CS_2. B) FTIR spectra of KCC-1 and KCC-1-NH-CS_2.

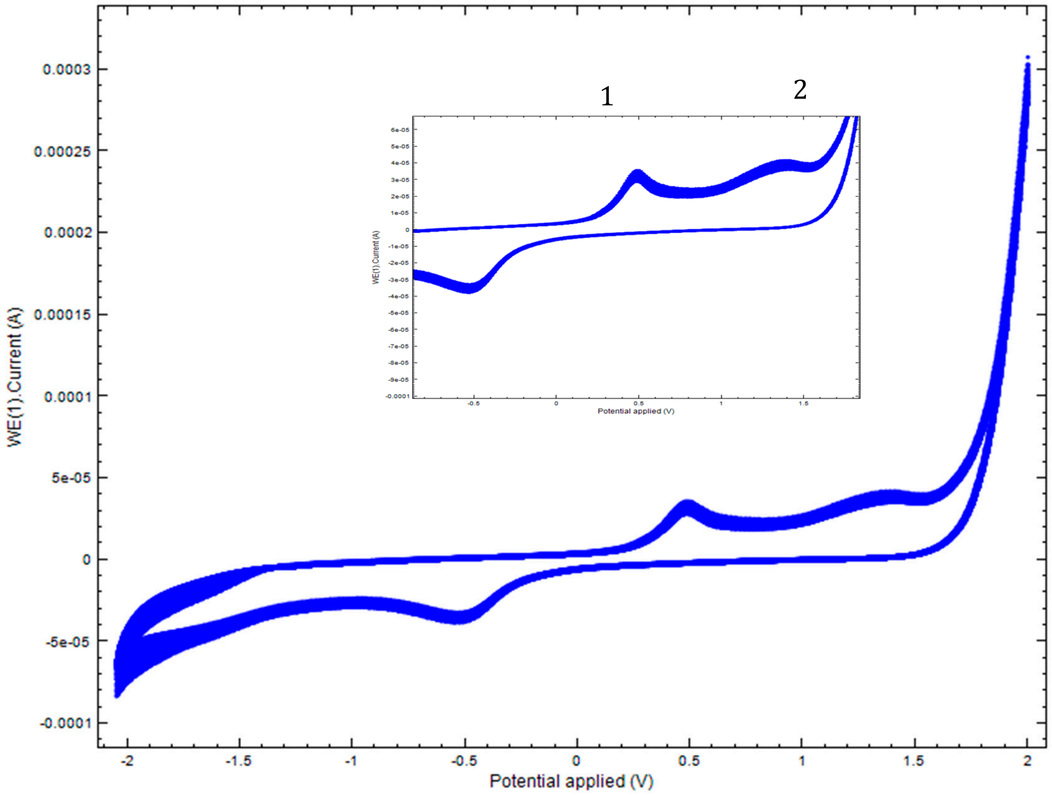

Figure S4. A) Electropolymerization of 6 mM β-CD in 0.05 M PBS (pH=4) as a supporting electrolyte on the surface of GCE. The cyclic voltammogram depicts gradual growth of β-CD after 40 successive cycles in the range of -2 to +2 V vs. Ag/AgCl with a scan rate of 0.07 V/s. Inset: three current peaks of *β*-CD electropolymerization.

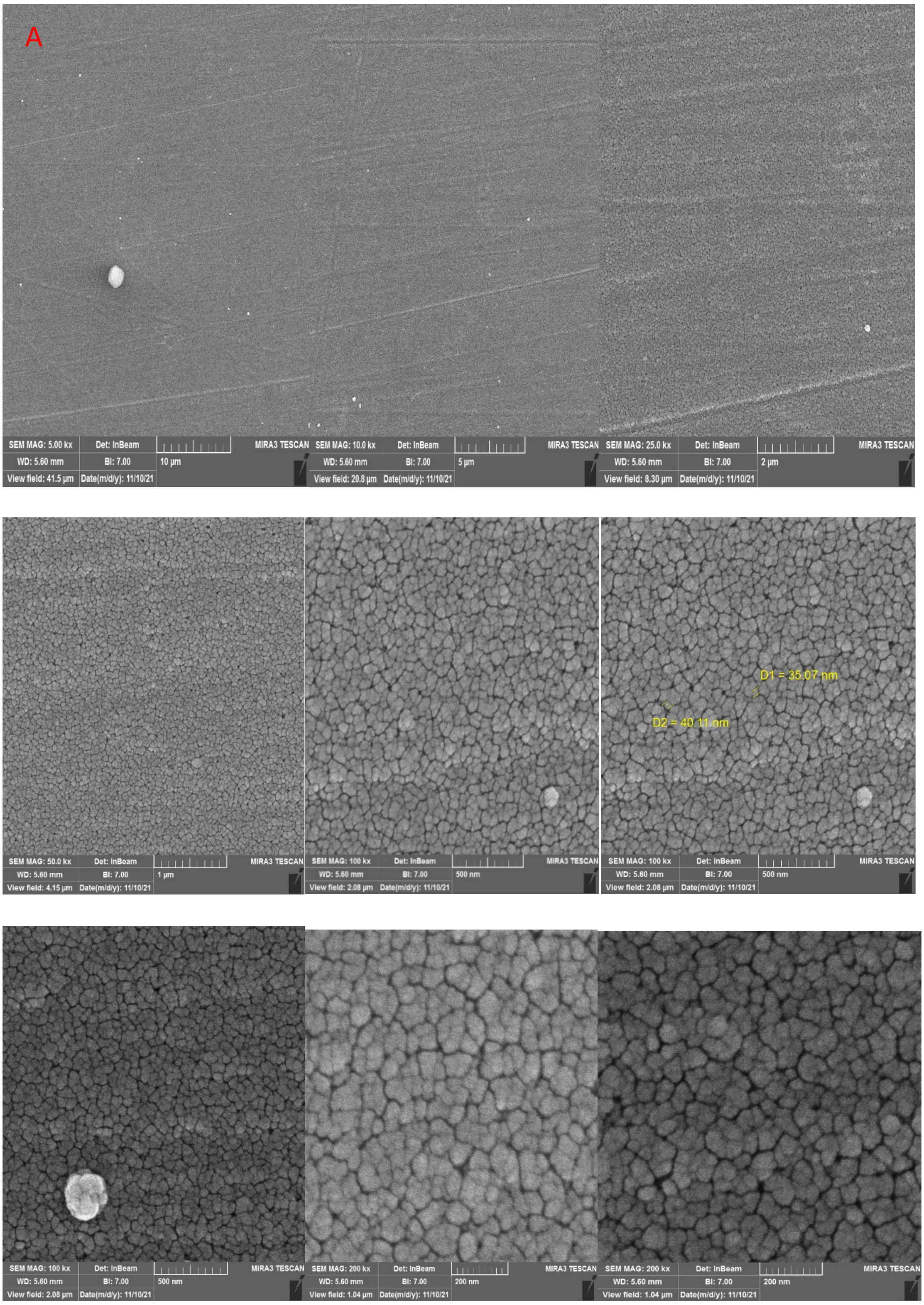

A
SEM MAG: 5.00 kx
Det: InBeam
WD: 5.60 mm
BI: 7.00
10 µm
View field: 41.5 µm
Date(m/d/y): 11/10/21
MIRA3 TESCAN
SEM MAG: 10.0 kx
Det: InBeam
WD: 5.60 mm
BI: 7.00
5 µm
View field: 20.8 µm
Date(m/d/y): 11/10/21
MIRA3 TESCAN
SEM MAG: 25.0 kx
Det: InBeam
WD: 5.60 mm
BI: 7.00
2 µm
View field: 8.30 µm
Date(m/d/y): 11/10/21
MIRA3 TESCAN
SEM MAG: 50.0 kx
Det: InBeam
WD: 5.60 mm
BI: 7.00
1 µm
View field: 4.15 µm
Date(m/d/y): 11/10/21
MIRA3 TESCAN
SEM MAG: 100 kx
Det: InBeam
WD: 5.60 mm
BI: 7.00
500 nm
View field: 2.08 µm
Date(m/d/y): 11/10/21
MIRA3 TESCAN
D1 = 35.07 nm
D2 = 40.11 nm
SEM MAG: 100 kx
Det: InBeam
WD: 5.60 mm
BI: 7.00
500 nm
View field: 2.08 µm
Date(m/d/y): 11/10/21
MIRA3 TESCAN
SEM MAG: 100 kx
Det: InBeam
WD: 5.60 mm
BI: 7.00
500 nm
View field: 2.08 µm
Date(m/d/y): 11/10/21
MIRA3 TESCAN
SEM MAG: 200 kx
Det: InBeam
WD: 5.60 mm
BI: 7.00
200 nm
View field: 1.04 µm
Date(m/d/y): 11/10/21
MIRA3 TESCAN
SEM MAG: 200 kx
Det: InBeam
WD: 5.60 mm
BI: 7.00
200 nm
View field: 1.04 µm
Date(m/d/y): 11/10/21
MIRA3 TESCAN

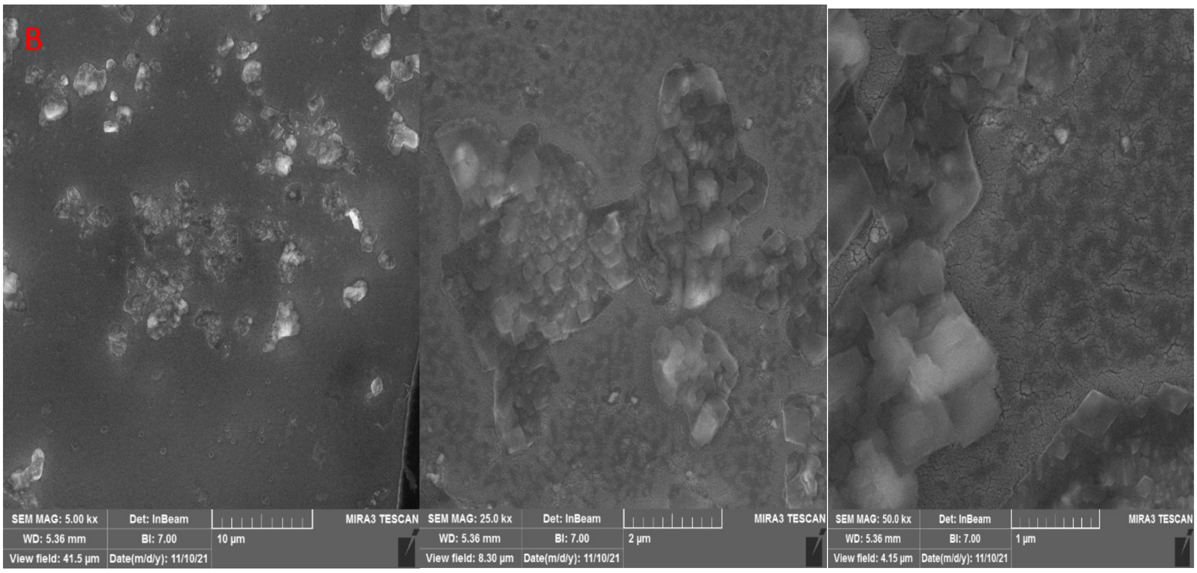
B
SEM MAG: 5.00 kx
Det: InBeam
10 μm
MIRA3 TESCAN
WD: 5.36 mm
BI: 7.00
View field: 41.5 μm
Date(m/d/y): 11/10/21
SEM MAG: 25.0 kx
Det: InBeam
2 μm
MIRA3 TESCAN
WD: 5.36 mm
BI: 7.00
View field: 8.30 μm
Date(m/d/y): 11/10/21
SEM MAG: 50.0 kx
Det: InBeam
1 μm
MIRA3 TESCAN
WD: 5.36 mm
BI: 7.00
View field: 4.15 μm
Date(m/d/y): 11/10/21

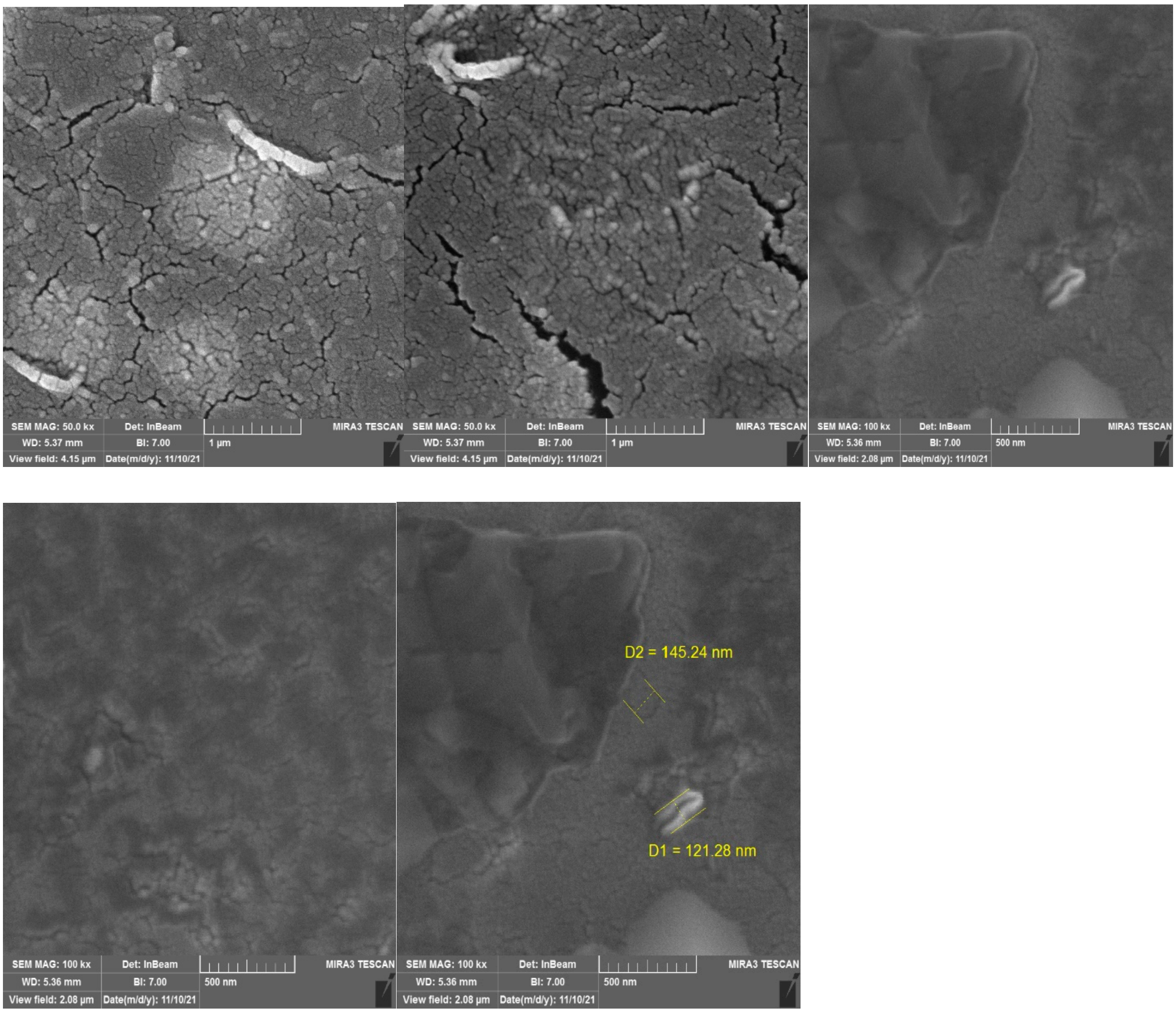
SEM MAG: 50.0 kx
Det: InBeam
MIRA3 TESCAN
WD: 5.37 mm
BI: 7.00
1 µm
View field: 4.15 µm
Date(m/d/y): 11/10/21
SEM MAG: 50.0 kx
Det: InBeam
MIRA3 TESCAN
WD: 5.37 mm
BI: 7.00
1 µm
View field: 4.15 µm
Date(m/d/y): 11/10/21
SEM MAG: 100 kx
Det: InBeam
MIRA3 TESCAN
WD: 5.36 mm
BI: 7.00
500 nm
View field: 2.08 µm
Date(m/d/y): 11/10/21
SEM MAG: 100 kx
Det: InBeam
MIRA3 TESCAN
WD: 5.36 mm
BI: 7.00
500 nm
View field: 2.08 µm
Date(m/d/y): 11/10/21
D2 = 145.24 nm
D1 = 121.28 nm
SEM MAG: 100 kx
Det: InBeam
MIRA3 TESCAN
WD: 5.36 mm
BI: 7.00
500 nm
View field: 2.08 µm
Date(m/d/y): 11/10/21

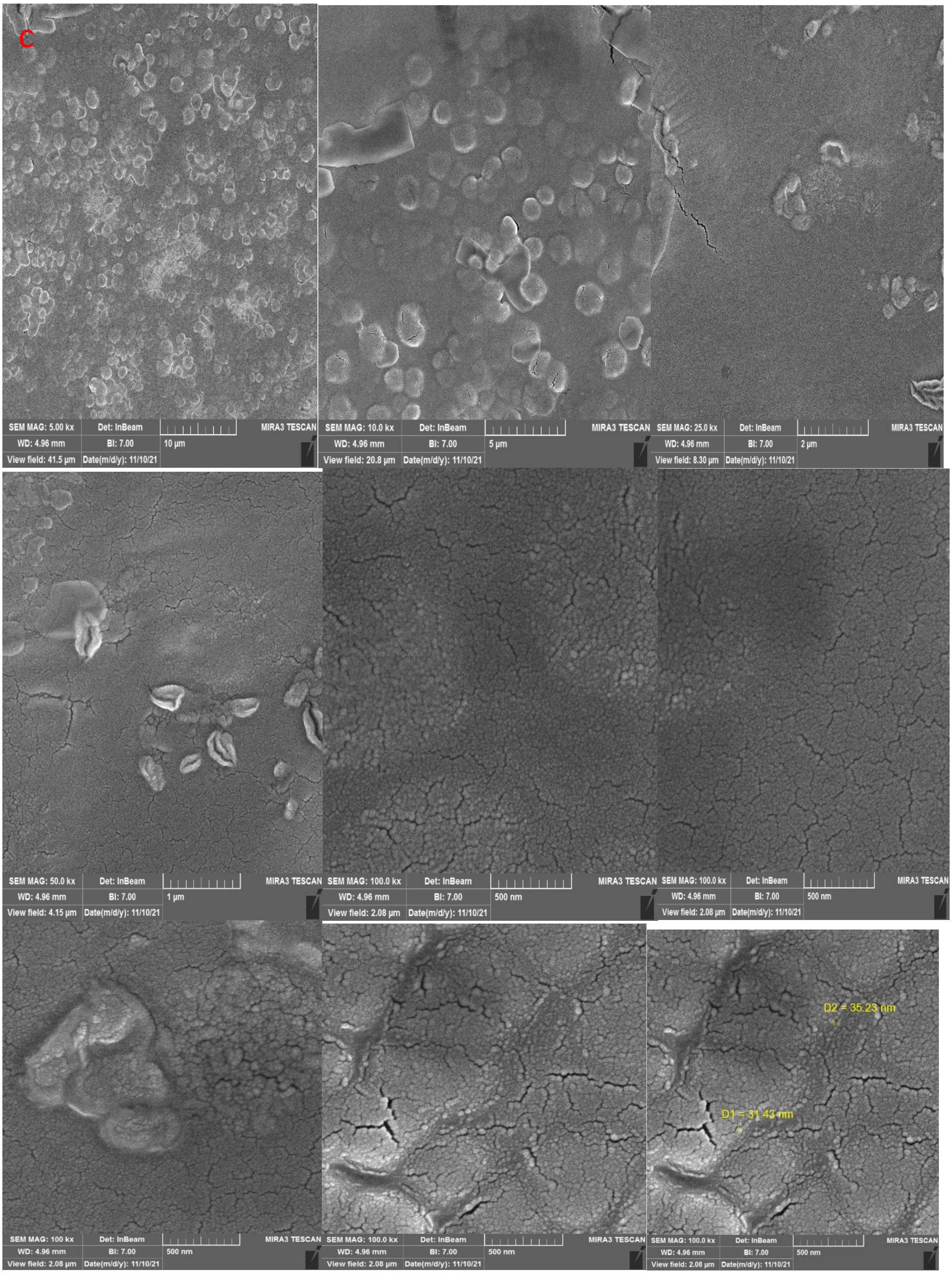

Figure S5. FE-SEM images of the sequential modified layers on GCE: A) β-CD, B) β-CD – KCC-1-NH-CS_2-Ab C) β-CD – KCC-1-NH-CS_2-Ab-BSA-Ag in different magnifications.

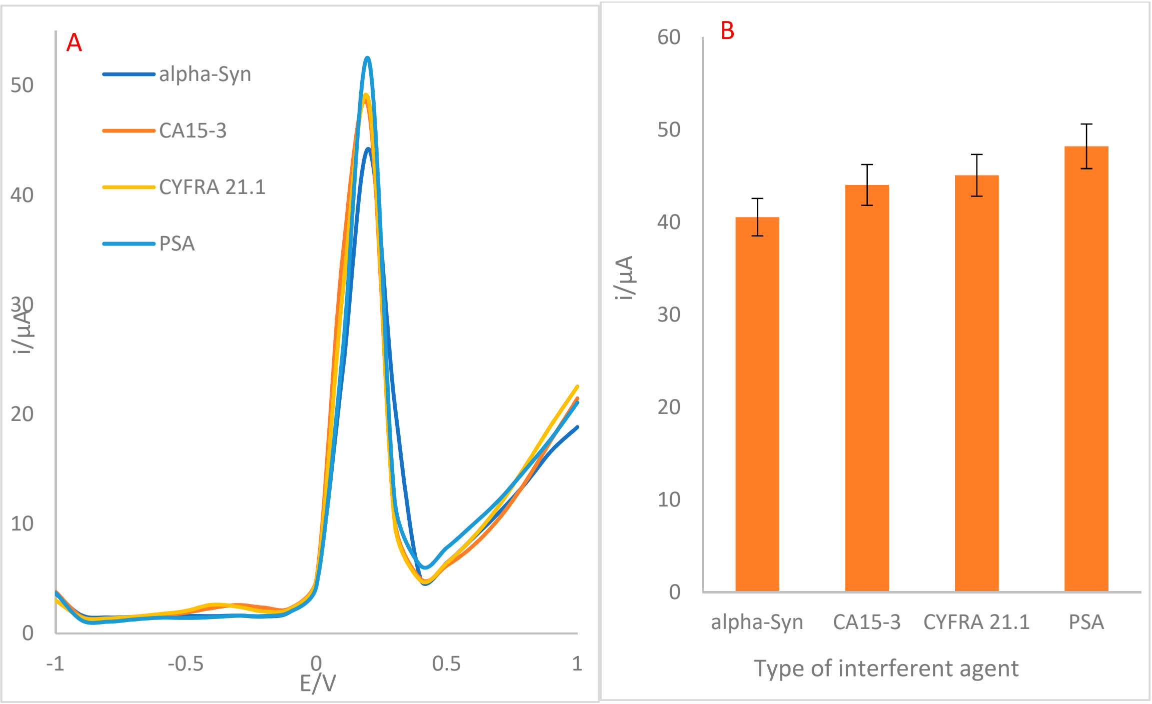

Figure S6. A) DPV of GCE-β-CD–KCC-1-NH-CS_2-Ab-BSA in the presence of α-syn and three other interferences. The supporting electrolyte was 0.05 M solution $[Fe(CN)_6]^{3-/4-}$/KCl; the potential range was from -1 to 1. B) comparison of α-syn and three other interferences peak current. (SD=2.28, n=4).

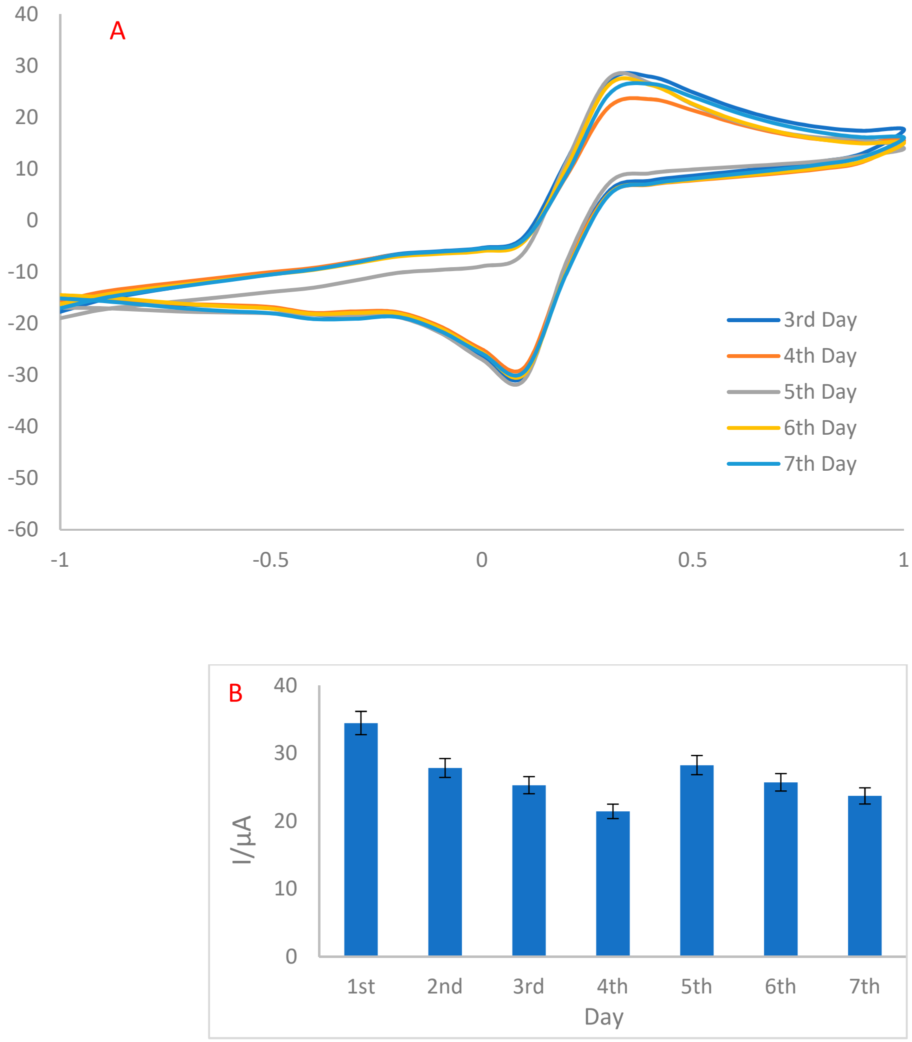

Figure S7. A) CV of GCE-P(β-CD) in 7 consecutive days. B) Comparison of 7 consecutive days' peak current intensity for GCE-P(β-CD) versus time of storage (SD=1.74, n=4).

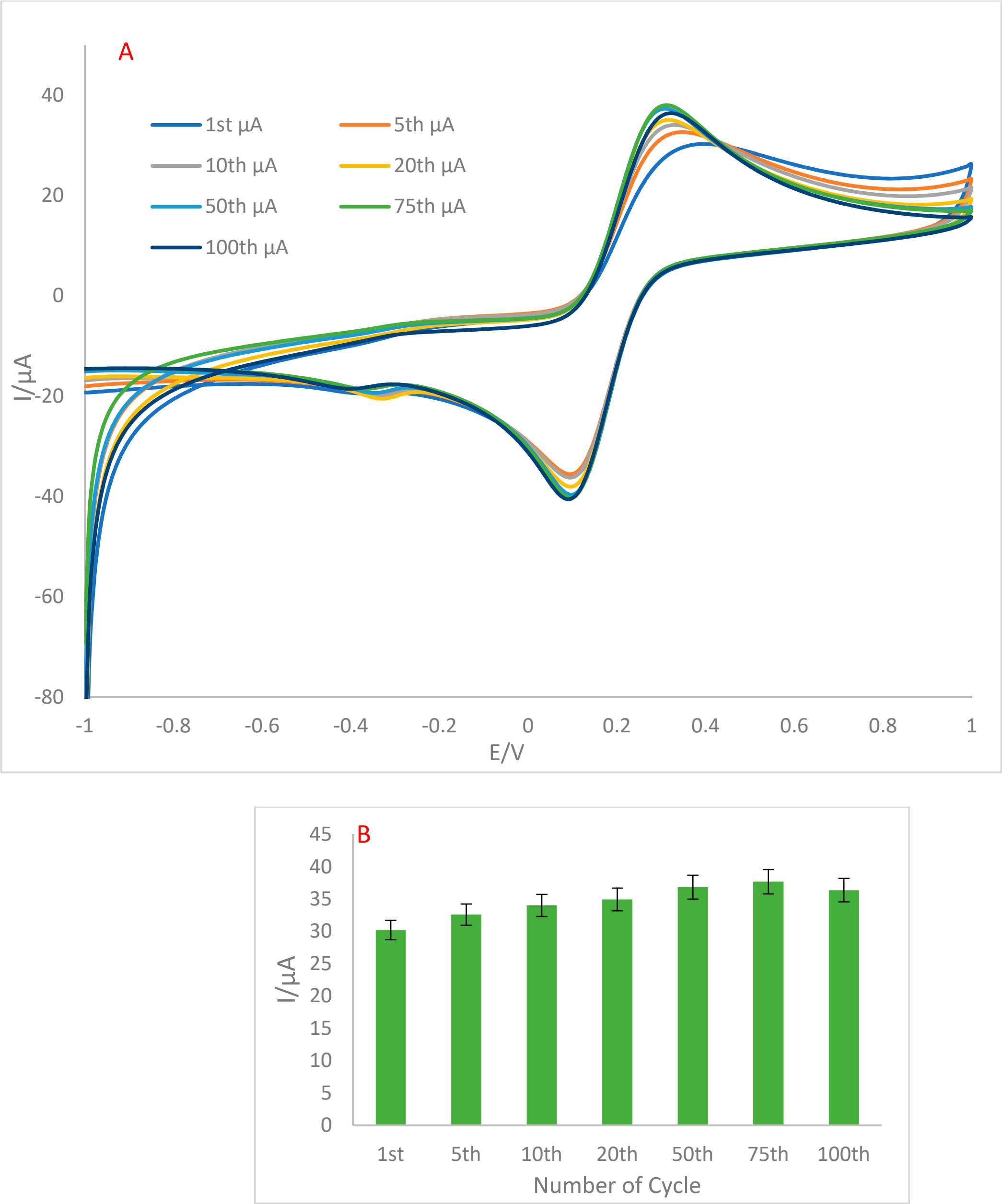

Figure S8. A) CV curves of GCE-P(β-CD) for the 1st, 5th, 10th, 20th, 50th, 75th, and 100th cycles.

B) Dependency of peak currents versus the number of cycles (SD=1.95, n=4).

Table S1. Electrochemical parameters of GCE-P(β-CD) and GCE (bare electrode).

DATA	GCE-P(β-CD)	GCE
α_n	0.59	0.33
D	3.16×10^{-5} cm^2 s^{-1}	1.17×10^{-6} cm^2 s^{-1}
A_{eas}	9.05×10^{-9} mol. cm^{-2}	2.11×10^{-10} mol. cm^{-2}
k_0	$8.8 \times 10^{+5}$ cm^3 mol^{-1}s^{-1}	$6.28 \times 10^{+4}$ cm^3 mol^{-1}s^{-1}

Table S2. Analytical parameters determined for calibration curves of α-Syn using engineered immunosensor.

Slop	Intercept	R^2	Linear range	LLOQ	Repeatability (RSD)	K_D[a](nM)
0.0096	4.896	0.9836	0.02 to 64 ng/mL	0.02 ng/mL	1.49%	2.36

[a] K_D dissociation constant of antigen-antibody complexes.
